# Discriminatory Target Learning: Mining Significant Dependence Relationships from Labeled and Unlabeled Data

**DOI:** 10.3390/e21050537

**Published:** 2019-05-26

**Authors:** Zhi-Yi Duan, Li-Min Wang, Musa Mammadov, Hua Lou, Ming-Hui Sun

**Affiliations:** 1 Key Laboratory of Symbolic Computation and Knowledge Engineering of Ministry of Education, Jilin University, Changchun 130012, China; 2 Faculty of Science, Engineering & Built Environment, Deakin University Geelong, Burwood, VIC 3125, Australia; 3 Changzhou College of Information Technology, Changzhou 213164, China; 4 College of Computer Science and Technology, Jilin University, Changchun 130012, China

**Keywords:** Bayesian network, discriminatory target learning, unlabeled instance

## Abstract

Machine learning techniques have shown superior predictive power, among which Bayesian network classifiers (BNCs) have remained of great interest due to its capacity to demonstrate complex dependence relationships. Most traditional BNCs tend to build only one model to fit training instances by analyzing independence between attributes using conditional mutual information. However, for different class labels, the conditional dependence relationships may be different rather than invariant when attributes take different values, which may result in classification bias. To address this issue, we propose a novel framework, called discriminatory target learning, which can be regarded as a tradeoff between probabilistic model learned from unlabeled instance at the uncertain end and that learned from labeled training data at the certain end. The final model can discriminately represent the dependence relationships hidden in unlabeled instance with respect to different possible class labels. Taking *k*-dependence Bayesian classifier as an example, experimental comparison on 42 publicly available datasets indicated that the final model achieved competitive classification performance compared to state-of-the-art learners such as Random forest and averaged one-dependence estimators.

## 1. Introduction

With the rapid development of computer technologies, business and government organizations create large amounts of data, which need to be processed and analyzed. Over the past decade, to satisfy the urgent need of mining knowledge hidden in the data, numerous machine learning models [1,2] (e.g., decision tree [3], Bayesian network [4,5], support vector machine [6] and Neural network [7]) have been proposed.

To mine all “right” knowledge that exist in a database, researchers mainly proposed two kinds of learning strategies to address this issue. (1) Increase structure complexity to represent more dependence relationships, e.g., convolutional neural network [8] and *k*-dependence Bayesian classifier (KDB) [9]. However, as structure complexity grows overfitting will inevitably appear, which will result in redundant dependencies and performance degradation. Sometimes the overly complex structures hide the internal working mechanism and make them criticized for being used as “black box”. (2) Build ensemble of several individual members having relatively simple network structure, e.g., Random forest [10] and averaged one-dependence estimators (AODE) [11]. Ensembles can generally perform better than any individual member. However, it is difficult or even impossible to give a clear semantic explanation of the combined result since the working mechanisms of individual members may differ greatly. In practice, people would rather use models with simple and easy-to-explain structures, e.g., decision tree [12] and Naive Bayes (NB) [13,14,15], although they may perform poorer.

Bayesian networks (BNs) have long been a popular medium for graphically representing the probabilistic dependencies, which exist in a domain. Recently, work in Bayesian methods for classification has grown enormously. Numerous Bayesian network classifiers (BNCs) [9,16,17,18,19,20] have been proposed to mine the significant dependence relationships implicated in training data. With solid theoretic support, they have strong potential to be effective for practical application in a number of massive and complex data-intensive fields such as medicine [21], astronomy [22], biology [23], and so on. A central concern for BNC is to learn conditional dependence relationships encoded in the network structure. Some BNCs, e.g., KDB, use conditional mutual information $I(X_{i};X_{j}|Y)$ to measure the conditional dependence relationships between $X_{i}$ and $X_{j}$, which is defined as follows [24],

(1) $$\begin{array}{ll} I(X_{i};X_{j}|Y) & =\sum_{x_{i}}\sum_{x_{j}}\sum_{y}P\left(x_{i},x_{j},y\right)log\frac{P(x_{i},x_{j}|y)}{P\left(x_{i}|y\right)P\left(x_{j}|y\right)}\\ & =\sum_{x_{i}}\sum_{x_{j}}\sum_{y}I\left(x_{i};x_{j}|y\right) \end{array}$$

**Figure 1:**
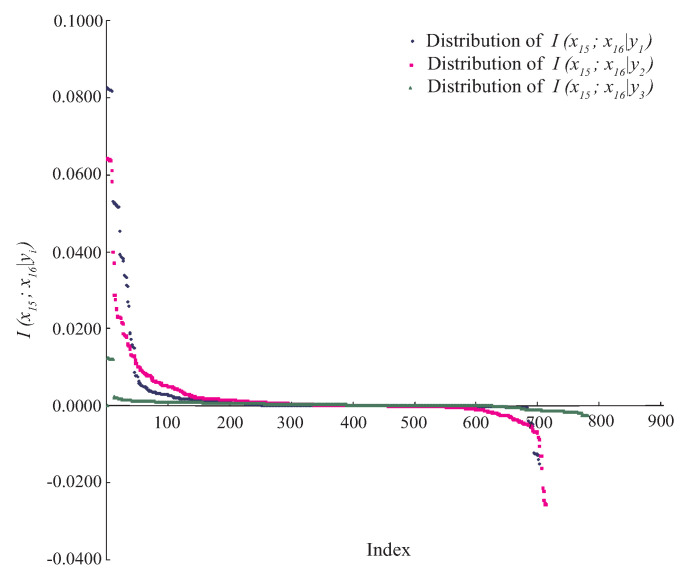
The distributions of $I(x_{15};x_{16}|y_{i})$ on Waveform dataset, where i∈{1,2,3}. The x-axis represents the index of each instance, the y-axis represents the value of $I(x_{15};x_{16}|y_{i})$.

For example, $I(X_{i};X_{j}|Y)=0$ indicates that attributes $X_{i}$ and $X_{j}$ are conditionally independent. However, in practice, for any specific event or data point, the situation will be much more complex. Taking Waveform dataset as an example, attributes $X_{15}$ and $X_{16}$ are conditionally dependent, since $I(X_{15};X_{16}|Y)>0$ always holds. Figure 1 shows the distributions of $I(x_{15};x_{16}|y_{i})$, where i∈{1,2,3}. As can be seen, there exist some positive values of $I(x_{15};x_{16}|y_{1})$ and $I(x_{15};x_{16}|y_{2})$. However, for the class label $y_{3}$, the negative or zero values of $I(x_{15};x_{16}|y_{3})$ have a high proportion among all values. That is, for different class labels, the conditional dependence relationships may be different rather than invariant when attributes take different values. We argue that most BNCs (e.g., NB and KDB), which build only one model to fit training instances, cannot capture this difference and cannot represent the dependence relationships flexibly, especially hidden in unlabeled instances.

The scientific data can be massive, and labeled training data may account for only a small portion. In this paper, we propose a novel learning framework, called discriminatory target learning, for achieving better classification performance and high-level of dependence relationships while not increasing structure complexity. KDB is taken as an example to illustrate the basic idea and prove the feasibility of discriminatory target learning. By redefining mutual information and conditional mutual information, we build a “precise” model kdb*_i_* for each unlabeled instance x with respect to class label $y_{i}$. The ensemble of kdb*_i_*, i.e., kdb^*e*^, can finely describe the dependency relationships hidden in x. The final ensemble of kdb^*e*^ and regular KDB can fully and discriminately describe the dependence relationships in training data and unlabeled instance.

The rest of the paper is organized as follows: Section 2 introduces some state-of-the-art BNCs. Section 3 introduces the basic idea of discriminatory target learning. Experimental study on 42 UCI machine learning datasets is presented in Section 4, including a comparison with seven algorithms. The final section draws conclusions and outlines some directions for further research.

## 2. Bayesian Network Classifiers

The structure of a BN on the random variables $\left\{X_{1},\cdots ,X_{n}\right\}$ is a directed acyclic graph (DAG), which represents each attribute in a given domain as a node in the graph and dependencies between these attributes as arcs connecting the respective nodes. Thus, independencies are represented by the lack of arcs connecting particular nodes. BNs are powerful tools for knowledge representation and inference under conditions of uncertainty. BNs were considered as classifiers only after the discovery of NB, a very simple kind of BN on the basis of conditional independence assumption. It is surprisingly effective and efficient for inference [5]. The success of NB has led to the research of Bayesian network classifiers (BNCs), including tree-augmented naive Bayes (TAN) [16], averaged one-dependence estimators (AODE) [18] and *k*-dependence Bayesian classifier (KDB) [9,17].

Let each instance **x** be characterized with *n* values $\{x_{1},\cdots ,$$x_{n}\}$ for attributes $\left\{X_{1},\cdots ,X_{n}\right\}$, and class label $y\in \{y_{1},\cdots ,$$y_{m}\}$ is the value of class variable *Y*. NB assumes that the predictive attributes are conditional independent of each other given the class label, that is

$$P\left(x_{1},\cdots ,x_{n}|y\right)=\prod_{i=1}^{n}P\left(x_{i}|y\right)$$

Correspondingly for any value pair of arbitrary two attributes $X_{i}$ and $X_{j}$, $P\left(x_{i},x_{j}|y\right)=P\left(x_{i}|y\right)P\left(x_{j}|y\right)$ always holds. From Equation (1) there will be $I(X_{i};X_{j}|Y)=0$ and this can explain why there exist no arc between attributes for NB. However, in the real world, it will be much more complex when considering different specific event or data point. We now formalize our notion of the spectrum of point dependency relationship in Bayesian classification.

**Definition** **1.** *For unlabeled data point $x=\{x_{1},\cdots ,x_{n}\}$, the conditional dependence between $X_{i}$ and $X_{j}\;\left(1\leq i,j\leq n\right)$ with respect to label y on point x is measured by pointwise y-conditional mutual information, which is defined as follows,*

(2) $$\begin{array}{cc} I(x_{i};x_{j}|y) & =P\left(x_{i},x_{j},y\right)\log\frac{P(x_{i},x_{j}|y)}{P\left(x_{i}|y\right)P\left(x_{j}|y\right)}\\ & =P\left(x_{i},x_{j},y\right)\log\frac{P(x_{i}|x_{j},y)}{P(x_{i}|y)} \end{array}$$

Equation (2) is a modified version of pointwise conditional mutual information that is applicable to labeled data point [25]. By comparing Equations (1) and (2), $I(X_{i};X_{j}|Y)$ is a summation of expected values of $I(x_{i};x_{j}|y)$ given all possible values of $X_{i},X_{j}$ and *Y*. The traditional BNCs, e.g., TAN and KDB, use $I(X_{i};X_{j}|Y)$ to roughly measure the conditional dependence between $X_{i}$ and $X_{j}$. $I(X_{i};X_{j}|Y)$ is non-negative, $I(X_{i};X_{j}|Y)>0$ iff $X_{i}$ and $X_{j}$ are conditionally dependent given *Y*. However, only considering $I(X_{i};X_{j}|Y)=0$ as the criterion for identifying the conditional independent relationship is too strict for BN learning, which may lead to classification bias, since $I(x_{i};x_{j}|y)\leq 0$ may hold for specific data point x. That may be the main reason why NB performs better in some research domains. To address this issue, in this paper $I(x_{i};x_{j}|y)$ is applied to measure the extent to which $X_{i}$ and $X_{j}$ are relatively conditionally dependent when $P\left(x_{i}|x_{j},y\right)>P\left(x_{i}|y\right)$ or relatively conditionally independent or irrelevant when $P\left(x_{i}|x_{j},y\right)<P\left(x_{i}|y\right)$, respectively.

**Definition** **2.** *For unlabeled data point $x=\{x_{1},\cdots ,x_{n}\}$ with respect to label y, if $I\left(x_{i};x_{j}|y\right)>0\;\left(1\leq i,j\leq n\right)$, then $X_{i}$ and $X_{j}$ are y-conditionally dependent on point x; if $I(x_{i};x_{j}|y)=0$, then they are y-conditionally independent on point x; and if $I(x_{i};x_{j}|y)<0$, then they are y-conditionally irrelevant on point x.*

TAN maintains the structure of NB and allows each attribute to have at most one parent. Then, the number of arcs encoded in TAN is n-1. During the constructing procedure of maximum weighted spanning tree, TAN sorts the arcs between arbitrary attributes $X_{i}$ and $X_{j}$ by comparing $I(X_{i};X_{j}|Y)$, and adds them in turn to the network structure if no cycle appears. KDB further relaxes NB’s independence assumption and can represent arbitrary degree of dependence while capturing much of the computational efficiency of NB. KDB first sorts attributes by comparing mutual information $I(X_{i};Y)$, which is defined as follows [24],

(3) $$I\left(X_{i};Y\right)=\sum_{x_{i}}\sum_{y}P\left(x_{i},y\right)log\frac{P(x_{i},y)}{P\left(x_{i}\right)P\left(y\right)}$$

Suppose the attribute order is $\left\{X_{1},\cdots ,X_{n}\right\}$. By comparing $I(X_{i};X_{j}|Y)$, $X_{i}$ select its parents, e.g., $X_{j}$, from attributes that ranks before it in the order. KDB requires that $X_{i}$ must have min(i-1,k) parents and there will exist min(i-1,k) arcs between $X_{i}$ and its parents. The number of arcs encoded in KDB is $nk-\frac{k^{2}}{2}-\frac{k}{2}$ and will grow as *k* grows. Thus, KDB can represent more dependency relationships than TAN. For TAN or KDB, they do not evaluate the extent to which the conditional dependencies are weak enough and should be neglected. They simply specify the maximum number of parents that attribute $X_{i}$ can have before structure learning. Some arcs corresponding to weak conditional dependencies will inevitably be added to the network structure. The prior and joint probabilities in Equations (1) and (3) will be estimated from training data as follows:

(4) $$\left\{\begin{array}{c} P\left(y\right)=\frac{1}{N}\mathrm{Count}\left(Y=y\right)\\ P\left(x_{j}\right)=\frac{1}{N}\mathrm{Count}\left(X_{j}=x_{j}\right)\\ P\left(x_{j},y\right)=\frac{1}{N}\mathrm{Count}\left(X_{j}=x_{j},Y=y\right)\\ P\left(x_{i},x_{j},y\right)=\frac{1}{N}\mathrm{Count}\left(X_{i}=x_{i},X_{j}=x_{j},Y=y\right) \end{array}\right.$$

where *N* is the number of training instances. Then, $P(x_{j}|y)$ and $P(x_{i},x_{j}|y)$ in Equations (1) and (3) can be computed as follows:

(5) $$\left\{\begin{array}{c} P\left(x_{j}|y\right)=\frac{P(x_{j},y)}{P(y)}\\ P\left(x_{i},x_{j}|y\right)=\frac{P(x_{i},x_{j},y)}{P(y)} \end{array}\right.$$

Sahami [9] suggested that, if *k* is large enough to capture all “right” conditional dependencies that exist in a database, then a classifier would be expected to achieve optimal Bayesian accuracy. However, as *k* grows, KDB will encode more weak dependency relationships, which correspond to smaller value of $I(X_{i};X_{j}|Y)$. That increases the risk of occurrence of negative values of $I(x_{i};x_{j}|y)$ and may introduce redundant dependencies, which will mitigate the positive effect from significant dependencies that correspond to positive values of $I(x_{i};x_{j}|y)$. On the other hand, conditional mutual information $I(X_{i};X_{j}|Y)$ cannot finely measure the conditional dependencies hidden in different data points. The arc $X_{i}\to X_{j}$ in BNC learned from training data corresponds to positive value of $I(X_{i};X_{j}|Y)$ and represents strong conditional dependence between $X_{i}$ and $X_{j}$. However, for specific labeled instance $d=\{x_{1},\cdots ,x_{n},y_{1}\}$, $I(x_{i};x_{j}|y_{1})\leq 0$ may hold. Then, $X_{i}$ and $X_{j}$ are $y_{1}$-conditionally independent or irrelevant on point d and the arc $X_{i}\to X_{j}$ should be removed. For unlabeled instance, the possible dependency relationships between nodes may differ greatly with respect to different class labels.

Thus, BNCs with highly complex network structure do not necessarily beat those with simple ones. The conditional dependencies hold for training data in general do not necessarily hold for each instance. BNCs should discriminate between conditionally dependent and irrelevant relationship for different data points. Besides, BNC should represent all possible spectrums of point dependency relationship that correspond to different class labels for dependence analysis.

## 3. Discriminatory Target Learning

**Figure 2:**
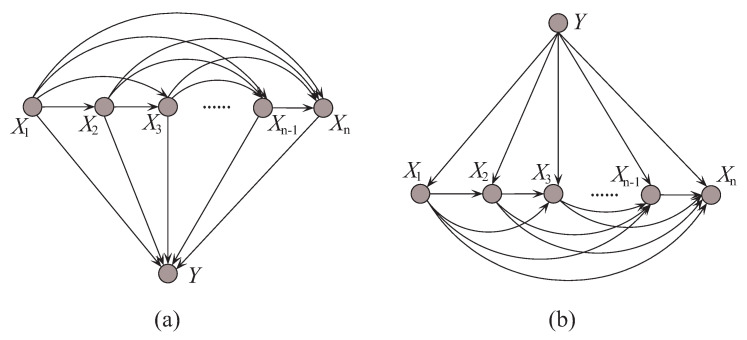
Example of (**a**) unrestricted BNC, and (**b**) restricted BNC.

In probabilistic classification, Bayes optimal classification suggests that, if we can determine the conditional probability distribution P(y|x) with true distribution available, where *y* is one of the *m* class labels and x is the *n*-dimensional data point $x=\{x_{1},x_{2},\cdots ,x_{n}\}$ that represents an observed instance, then we could achieve the theoretically optimal classification. P(y|x) can be described in an unrestricted Bayesian network, as shown in Figure 2a. By applying arc reversal, Shachter [26] proposed to produce the equivalent dependence structure, as shown in Figure 2b. The problem is reduced to estimating the conditional probability P(x|y). Figure 2a,b represents two inference processes that run in the opposite directions. Figure 2a indicates the causality that runs from the state of $\left\{X_{1},\cdots ,X_{n}\right\}$ (the cause) to the state of *Y* (the effect). In contrast, if the causality runs in the opposite direction as shown in Figure 2b and the state of *Y* (the effect) is uncertain, the dependencies between predictive attributes (the causes) should be tuned to match with different states of *Y*. That is, the restricted BNC shown in Figure 2b presupposes the class label first and then the conditional dependencies between attributes can verify the presupposition.

**Figure 3:**
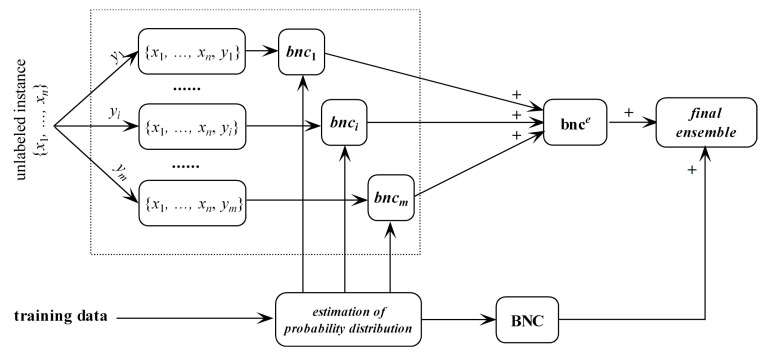
The framework of discriminatory target learning.

For different class labels or presuppositions, the conditional dependencies should be different. It is not reasonable that, no matter what the effect (class label) is, the relationships between causes (predictive attributes) remain the same. Consider an unlabeled instance $x=\{x_{1},\cdots ,x_{n}\}$; if $I(x_{i};x_{j}|y)>0$, then the conditional dependence between $X_{i}$ and $X_{j}$ on data point x with respect to class label *y* is reasonable, otherwise it should be neglected. Since the class label for x is uncertain and there are *m* labels available, we take x as the target and learn an ensemble of *m* micro BNCs, i.e., bnc^*e*^ = {bnc_1_, ⋯, bnc*_m_*}, each of them fully describes the conditional dependencies between attribute values in x with respect to different class labels. The linear combiner is used for models that output real-valued numbers, thus is applicable for bnc^*e*^. The ensemble probability estimate for bnc^*e*^ is,

(6) $$\hat{P}\left(y_{i}\right|x,{\mathrm{bnc}}^{e})=\frac{P(y_{i},x|{\mathrm{bnc}}_{i})}{\sum_{i=1}^{m}P\left(y_{i},x|{\mathrm{bnc}}_{i}\right)}.$$

bnc^*e*^ may overfit the unlabeled instance and underfit training data. In contrast, regular BNC learned from training data may underfit the unlabeled instance. Thus, they are complementary in nature. After training bnc^*e*^ and regular BNC, the final ensemble that estimates the class membership probabilities by averaging both predictions will be generated. The framework of discriminatory target learning is shown in Figure 3.

Because in practice it is hardly possible to find the true distribution of P(x|y) from data, KDB approximates the estimation of P(x|y) by allowing for the modeling of arbitrarily complex dependencies between attributes. The pseudocode of KDB is shown in Algorithm 1.

**Algorithm 1** Structure learning of KDB.

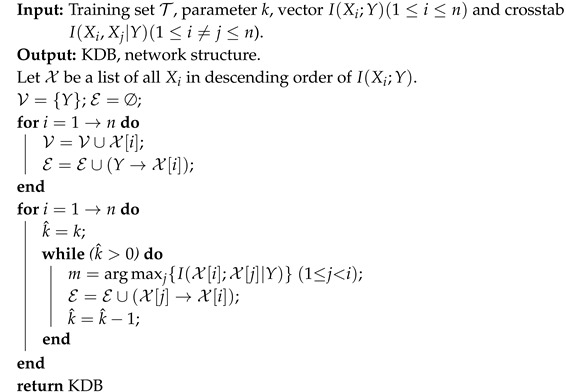



From the definition of $I(X_{i};Y)$ in Equation (3), we can have

(7) $$\begin{array}{cc} I(X_{i};Y) & =\sum_{y}\sum_{x_{i}}P\left(x_{i},y\right)log\frac{P(x_{i},y)}{P\left(x_{i}\right)P\left(y\right)}\\ & =\sum_{y}\sum_{x_{i}}P\left(x_{i},y\right)log\frac{P(y|x_{i})}{P(y)} \end{array}$$

**Definition** **3.** *For unlabeled data point $x=\{x_{1},\cdots ,x_{n}\}$, the dependence between $x_{i}\;\left(1\leq i\leq n\right)$ and any given label y is measured by pointwise y-mutual information, which is defined as follows,*

(8) $$I\left(x_{i};y\right)=P\left(x_{i},y\right)log\frac{P(x_{i},y)}{P\left(x_{i}\right)P\left(y\right)}=P\left(x_{i},y\right)log\frac{P(y|x_{i})}{P(y)}.$$

Equation (8) is a modified version of pointwise mutual information that is applicable to labeled data point [25]. The prior and joint probabilities in Equations (2) and (8) will be estimated as follows

(9) $$\left\{\begin{array}{c} \hat{P}\left(y\right)=\frac{1}{N+1}\left[\mathrm{Count}\left(Y=y\right)+\frac{1}{m}\right]\\ \hat{P}\left(x_{j}\right)=\frac{1}{N+1}\left[\mathrm{Count}\left(X_{j}=x_{j}\right)+\frac{1}{m}\right]\\ \hat{P}\left(x_{j},y\right)=\frac{1}{N+1}\left[\mathrm{Count}\left(X_{j}=x_{j},Y=y\right)+\frac{1}{m}\right]\\ \hat{P}\left(x_{i},x_{j},y\right)=\frac{1}{N+1}\left[\mathrm{Count}\left(X_{i}=x_{i},X_{j}=x_{j},Y=y\right)+\frac{1}{m}\right] \end{array}\right.$$

Conditional probabilities in Equations (2) and (8) can be estimated by:

(10) $$\left\{\begin{array}{c} \hat{P}\left(x_{j}|y\right)=\frac{\hat{P}\left(x_{j},y\right)}{\hat{P}\left(y\right)}\\ \hat{P}\left(x_{i},x_{j}|y\right)=\frac{\hat{P}\left(x_{i},x_{j},y\right)}{\hat{P}\left(y\right)}\\ \hat{P}\left(y|x_{i}\right)=\frac{\hat{P}\left(x_{i},y\right)}{\hat{P}\left(x_{i}\right)} \end{array}\right.$$

Similar to the Laplace correction [27], the main idea behind Equation (9) is equivalent to creating a “pseudo” training set P by adding to the training data a new instance $\left\{x_{1},\cdots ,x_{n}\right\}$ with multi-label by assuming that the probability that this new instance is in class *y* is 1/m for each $y\in \{y_{1},\cdots ,y_{m}\}.$

**Definition** **4.** *For unlabeled data point $x=\{x_{1},\cdots ,x_{n}\}$ with respect to label y, if $I\left(x_{i};y\right)>0\;\left(1\leq i\leq n\right)$, then $X_{i}$ is y-dependent on point x; if $I(x_{i};y)=0$, then $X_{i}$ is y-independent on point x; and if $I(x_{i};y)<0$, then $X_{i}$ is y-irrelevant on point x.*

KDB uses $I(X_{i};Y)$ to sort the attributes and $I(X_{i};X_{j}|Y)$ to measure the conditional dependence. Similarly, for unlabeled instance $x=\{x_{1},\cdots ,x_{n}\}$, the corresponding micro KDB with respect to class label $y_{t}$, called kdb*_t_*, uses $I(x_{i};y_{t})$ (see Equation (8)) to sort the attribute values and $I(x_{i};x_{j}|y_{t})$ (see Equation (2)) to measure the conditional dependence. The learning procedure of kdb*_t_* is shown in Algorithm 2.    

**Algorithm 2** Structure learning of kdb*_t_* with respect to class label $y_{t}$.

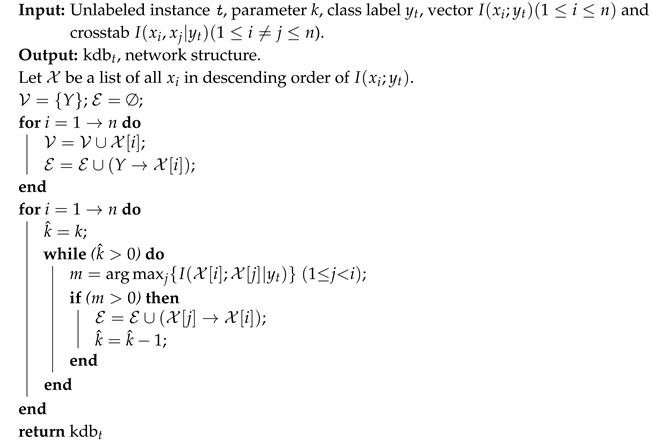



Breiman [28] revealed that ensemble learning brings improvement in accuracy only to those “unstable” learning algorithms, in the sense that small variations in the training set would lead them to produce very different models. bnc^*e*^ is obviously an example of such learners. For individual members of kdb^*e*^, the difference in network structure is the result of change of $I(x_{i};y)$ or $I\left(x_{i};x_{j}|y\right)\;\left(1\leq i\neq j\leq n\right)$, or, more precisely, the conditional probability defined in Equations (2) and (8). Given unlabeled instance $x=\{x_{1},\cdots ,x_{n}\}$ and binary class labels $y_{1}$ and $y_{2}$, if $I(x_{i};y_{1})>0$, i.e., $P\left(y_{1}|x_{i}\right)>P\left(y_{1}\right)$, then $X_{i}$ is $y_{1}$-dependent on x. Because $P\left(y_{2}\right)=1-P\left(y_{1}\right)$ and $P\left(y_{2}|x_{i}\right)=1-P\left(y_{1}|x_{i}\right),$ we have

(11) $$\begin{array}{cc} P\left(y_{1}|x_{i}\right)>P\left(y_{1}\right) & \Rightarrow 1-P\left(y_{1}|x_{i}\right)<1-P\left(y_{1}\right)\\ & \Rightarrow P\left(y_{2}|x_{i}\right)<P\left(y_{2}\right) \end{array}$$

and

(12) $$I\left(x_{i};y_{2}\right)=P\left(x_{i},y_{2}\right)log\frac{P(y_{2}|x_{i})}{P(y_{2})}<0$$

**Figure 4:**
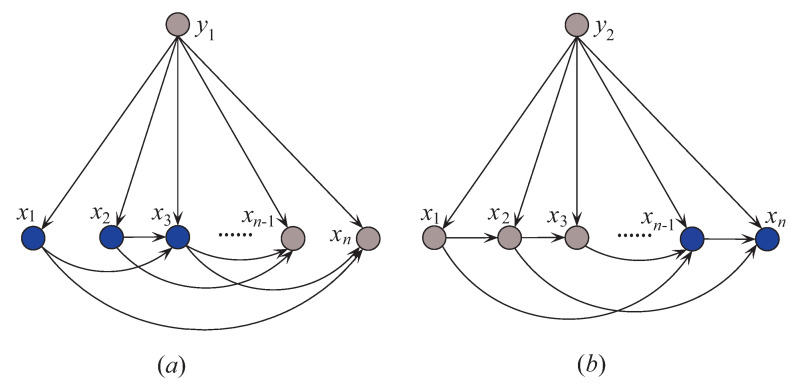
Example of (**a**) kdb_1_, and (**b**) kdb_2_.

Thus, $X_{i}$ is $y_{2}$-irrelevant on x. $X_{i}$ plays totally different roles in the relationships with different class labels on the same instance. Supposing that before small variations in the training set $I(x_{i};y_{1})>0$ and after that $I(x_{i};y_{1})<0$, the attribute values will be resorted and correspondingly the network structures of kdb_1_ and kdb_2_ for x will change greatly. The sensitivity to the variation makes kdb^*e*^ finely describe the dependencies hidden in x. Figure 4 shows examples of kdb_1_ and kdb_2_ corresponding to class labels $y_{1}$ and $y_{2}$, respectively. If the decision of the final ensemble is $y_{1}$, then we will use Figure 4a for dependence analysis. Otherwise, we will use Figure 4b instead. The attribute values annotated in black correspond to positive values of $I\left(x_{i};y_{t}\right)(t=1$ or 2) and they should be focused on.

KDB requires training time complexity of $O(n^{2}Nmv^{2})$ (dominated by the calculations of $I(X_{i};X_{j}|Y)$) and classification time complexity of $O(n^{2}Nm)$ [9] for classifying a single unlabeled instance, where *n* is the number of attributes, *N* is the number of data instances, *m* is the number of class labels, and *v* is the maximum number of discrete values that an attribute may take. Discriminatory target learning requires no additional training time, thus the training time complexity of final ensemble is the same as that of regular KDB. At classification time it requires $O(n^{2}Nm)$ to calculate $I(x_{i};x_{j}|y)$, and the same time complexity for classifying a single unlabeled instance.

## 4. Experiments and Results

We compared the performance of our proposed methods kdb^*e*^ and KDB^*e*^ with several state-of-the-art classifiers. We analyzed the performance in terms of zero-one loss, root mean square error (RMSE), bias and variance on 42 natural domains from the UCI Machine Learning Repository [29]. These datasets are described in Table 1, in ascending order of number of instances. The structure of this section is as follows: we discuss our experimental methodology and evaluation function in details in Section 4.1. Section 4.2 includes comparisons with three classic single-structure BNCs, namely NB, TAN and KDB, as well as one ensemble BNC: AODE. Then, in Section 4.3, KDB^*e*^ is compared with Random Forest with 100 decision trees. Section 4.4 presents a global comparison of all learners considered by applying the Friedman and Nemenyi tests.

### 4.1. Experimental Methodology and Evaluation Function

The experiments for all BNCs used C++ software (NetBeans 8.0.2) specially designed to deal with classification problems. Each algorithm was tested on each dataset using 10-fold cross validation. All experiments were conducted on a desktop computer with an Intel(R) Core(TM) i3-6100 CPU @ 3.70 GHz, 64 bits and 4096 MB of memory(Dell Vostro 2667, Changchun, China).

- Win/Draw/Lose (W/D/L) Record: When two algorithms were compared, we counted the number of datasets for which one algorithm performed better, equally well or worse than the other on a given measure. We considered there exists a significant difference if the output of a one-tailed binomial sign test was less than 0.05.
- Missing Values: Missing values for qualitative attributes were replaced with modes, and those for quantitative attributes were replaced with means from the training data.
- Numeric Attributes: For each dataset, we used MDL (Minimum Description Length) discretization [30] to discretize numeric attributes.
- Dataset Sizes: Datasets were categorized in terms of their sizes. That is, datasets with instances <1000, ≥1000 and <10,000, ≥10,000 were denoted as small size, medium size and large size, respectively. We report results on these sets to discuss suitability of a classifier for datasets of different sizes.
- Zero-one loss: Zero-one loss can be used to measure the extent to which a learner correctly identifies the class label of an unlabeled instance. Supposing *y* and $\hat{y}$ are the true class label and that generated by a learning algorithm, respectively, given *M* unlabeled test instances, the zero-one loss function is defined as
  $$\xi \left(y,\hat{y}\right)=\frac{\sum_{i=1}^{M}1-\varrho \left(y_{i},\hat{y_{i}}\right)}{M},$$
  where $\varrho (y_{i},\hat{y_{i}})=1$ if $y_{i}=\hat{y_{i}}$ and 0 otherwise.
- Bias and variance: The bias-variance decomposition proposed by Kohavi and Wolpert [31] provides valuable insights into the components of the zero-one loss of learned classifiers. Bias measures how closely the classifier can describe the decision boundary, which is defined as
  $$bias=\frac{1}{2}\sum_{\hat{y},y\epsilon Y}{\left[P\left(\hat{y}|x\right)-P\left(y|x\right)\right]}^{2},$$
  where x is the combination of any attribute value. Variance measures the sensitivity of the classifier to variations in the training data, which is defined as
  $$variance=\frac{1}{2}\left[1-\sum_{\hat{y}\epsilon Y}P{\left(\hat{y}|x\right)}^{2}\right].$$
- RMSE: For each instance, RMSE accumulates the squared error, where the error is the difference between 1.0 and the probability estimated by the classifier for the true class for the instance, and then computes the squared root of the mean of the sum, which is defined as
  $$RMSE=\sqrt{\frac{1}{s}\sum_{i=1}^{s}{\left(1-P\left(\hat{y}|x\right)\right)}^{2}},$$
  where *s* is the sum of training instances.

### 4.2. KDB^e^ Versus Classic BNCs

We compared KDB^*e*^ with several classic BNCs, namely NB, TAN, KDB and AODE. Sahami [9] proposed the notion of *k*-dependence BNC, which allows each attribute $X_{i}$ to have a maximum of *k* attributes as parents. NB and TAN are, respectively, 0-dependence and 1-dependence BNCs. To clarify the effect of dependence complexity, we set k=2 for both KDB and KDB^*e*^.

#### 4.2.1. Zero-One Loss and RMSE Results

The detailed results in terms of zero-one loss and RMSE are shown in Table A1 and Table A2 in Appendix A, respectively. Table 2 and Table 3 show W/D/L records summarizing the relative zero-one loss and RMSE of different BNCs. When k=2, NB, TAN and KDB can, respectively, represent 0, n-1 and 2n-3 conditional dependencies, where *n* is the number of predictive attributes. As shown in Table 1, since n>3 holds for all datasets, 2n-3>n-1 also holds. Thus, KDB can represent the largest number of dependencies among all. With respect to zero-one loss, NB represents no conditional dependencies due to its independence assumption and performed the worst in general. As the dependence degree or structure complexity increased, KDB was competitive compared to NB and TAN. AODE performed better than the other single-structure BNCs due to its ensemble mechanism. Surprisingly, kdb^*e*^ had significantly better zero-one loss performance than NB, TAN and KDB. When discriminatory target learning was introduced for discovery of dependencies that exist in different unlabeled instances, the final ensemble KDB^*e*^ could possess significant advantage over other classifiers. For example, KDB^*e*^ beat KDB in 26 domains and lost only in three in terms of zero-one loss. RMSE-wise, KDB^*e*^ still performed the best. For instance, KDB^*e*^ enjoyed a significant advantage over TAN (20/19/3). When compared to KDB, KDB^*e*^ also achieved superior performance, with 17 wins and 5 losses.

**Figure 5:**
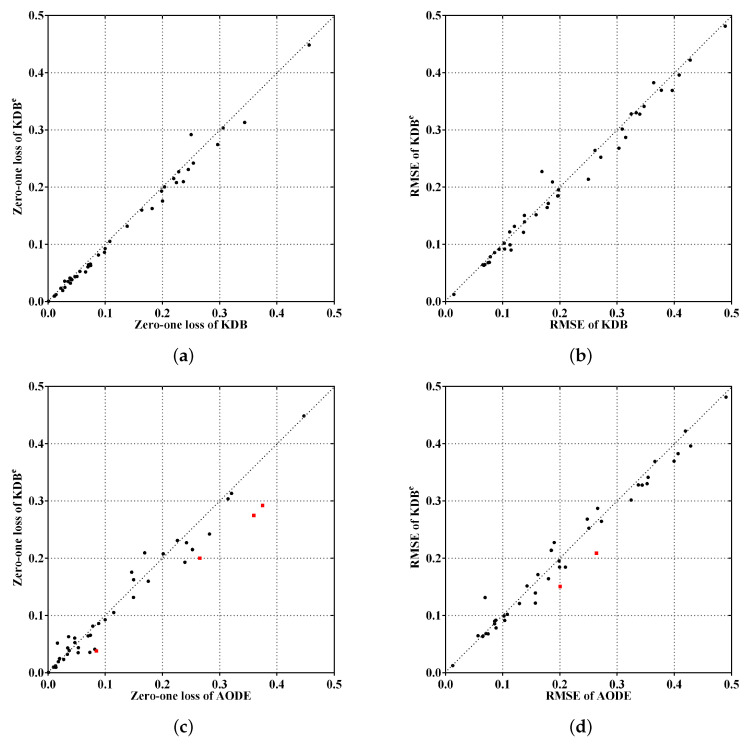
Scatter plot of zero-one loss and RMSE comparisons for KDB^*e*^, KDB and AODE.

To make the experimental results more intuitive, from the viewpoints of the ensemble mechanism and structure complexity, Figure 5a,c shows the comparisons of KDB^*e*^, KDB and AODE in terms of zero-one loss, whereas Figure 5b,d shows the comparisons for RMSE. The red squared symbols are used to indicate significant advantages of KDB^*e*^ over the other BNCs. In Figure 5a,b, only two points are far above the diagonal line, thus the negative effect caused by discriminatory target learning was negligible. In contrast, many more points are below the diagonal line, which means that discriminatory target learning worked effectively in most cases. A notable case is Waveform dataset, where discriminatory target learning helped to substantially reduce classification error, such as the reduction from 0.0256 to 0.0193 for zero-one loss and from 0.1145 to 0.0901 for RMSE. When comparing KDB^*e*^ with AODE, it can be seen in Figure 5c,d that there are still many points below the diagonal line, which means that KDB^*e*^ enjoyed a significant advantage over AODE. For example, a notable case is our largest dataset Localization, where the zero-one loss of KDB^*e*^ (0.2743) was much lower than that of AODE (0.3596).

#### 4.2.2. Bias and Variance Results

The detailed results in terms of bias and variance are shown in Table A3 and Table A4 in Appendix A, respectively. The W/D/L records with respect to bias and variance results are shown in Table 4 and Table 5, respectively. We can observe in Table 4 that ensemble classifiers, i.e., AODE and kdb^*e*^, performed better than TAN but worse than KDB, although these results were not always statistically significant. NB still performed the worst. High-dependence structure or ensemble construction strategy could help reduce the bias. Jointly applying both helped KDB^*e*^ reduce bias significantly. For example, KDB^*e*^ performed better than TAN (26/9/7) and KDB (11/27/4).

In terms of variance, since the network structures of NB and AODE are definite and irrelevant to the variation of the training data, the independence assumption helped reduce the variance significantly. KDB was the most sensitive to the variation in training data among all classifiers. As discussed in Section 3, discriminatory target learning made kdb^*e*^ underfit training data and overfit the unlabeled instance. When kdb^*e*^ was integrated with regular KDB, discriminatory target learning helped to reduce the variance and the final ensemble classifier, i.e., KDB^*e*^, performed the best only after NB and AODE.

#### 4.2.3. Time Comparison

**Figure 6:**
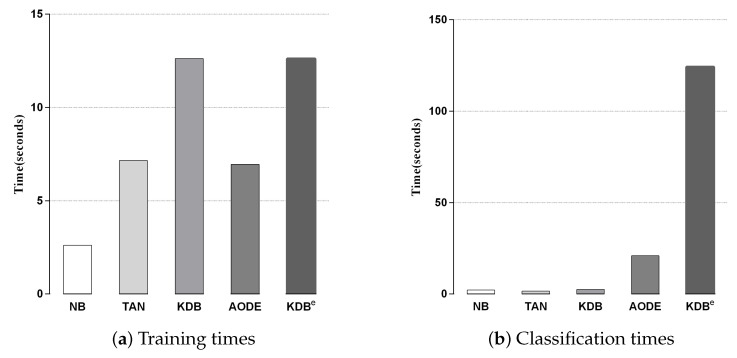
Training and classification time comparisons for BNCs.

We compared KDB^*e*^ with the other classic BNCs in terms of training and classification time. Since kdb^*e*^ is a part of KDB^*e*^, we removed it in this experiment. Figure 6a,b shows the training and classification time comparisons for all BNCs. Each bar represents the sum of time on 42 datasets in a 10-fold cross-validation experiment. No parallelization techniques were used in any case. As discussed in Section 3, discriminatory target learning requires no additional training time, thus the training time complexity of KDB^*e*^ was the same as that of regular KDB. Due to the structure complexity, KDB^*e*^ and KDB required a bit more time for training than the other BNCs. With respect to classification time, KDB^*e*^ took a little more time than the other BNCs. The reason lies in that KDB^*e*^ learned kdb^*e*^ for each unlabeled test instance, while the other BNCs only needed to directly calculate the joint probabilities. In general, discriminatory target learning helped to significantly improve the classification performance of its base classifier at the cost of a small increase in time consumption, which is perfectly acceptable.

### 4.3. KDB^e^ Versus Random Forest

To further illustrate the performance of our proposed discriminatory target learning framework, we compared KDB^*e*^ with a powerful learner, i.e., Random forest.Random forest (RF) is a combination of decision tree predictors, where each tree is trained on data selected at random but with replacement from the original data [10]. As the number of trees in the forest becomes large, the classification error for forests tends to converge to a limit. RF is an effective tool in prediction. RF can process high-dimensional data (that is, data with a lot of features) without making feature selection. Furthermore, due to the random mechanism, RF has the capacity to deal with imbalanced datasets or data with numerous missing values. Moreover, the framework in terms of strength of the individual predictors and their correlations gives insight into the ability of the RF to predict [10]. Because of its high classification accuracy, RF has been applied to many scientific fields, e.g., ecology and agriculture [32]. In our experiment, RF with 100 decision trees was used. The detailed results of RF in terms of zero-one loss, RMSE, bias and variance can be found in Table A1, Table A2, Table A3 and Table A4 in Appendix A, respectively. Table 6 shows the W/D/L records with different dataset sizes. When zero-one loss was compared, KDB^*e*^ won more frequently than RF, especially on small and medium datasets. The results indicate 10/4/3 on small datasets and 7/4/4 on medium datasets. The reason may lie in that 100 decision trees are complex and tend to overfit the training data. RMSE-wise, KDB^*e*^ also performed better than RF, which is shown as 16 wins and 11 losses. Bias and variance comparison of KDB^*e*^ and RF (Table 6) suggested that KDB^*e*^ is a low variance and high bias classifier. One can expect it to work extremely well on small and medium datasets. This is evident in Table 6 showing the zero-one loss and RMSE comparisons. KDB^*e*^ beat RF on 26 datasets and lost on 12 datasets with respect to variance. Thus, the advantages of KDB^*e*^ over RF in terms of zero-one loss and RMSE could be attributed to the change in variance. Since the variance term increased as the algorithm became more sensitive to the change in labeled training data, obviously, discriminatory target learning helped to alleviate the negative effect caused by overfitting.

**Figure 7:**
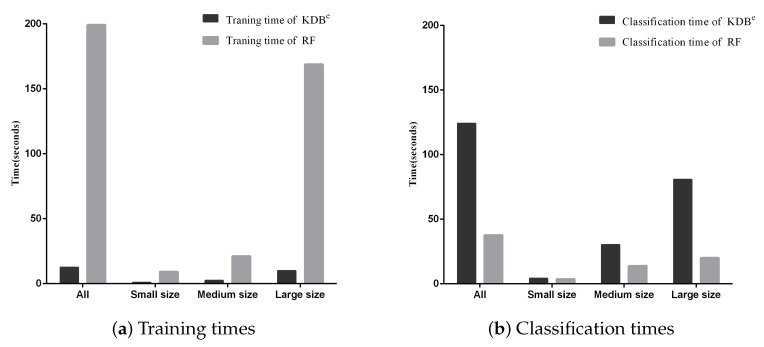
Training and classification time comparisons between KDB^*e*^ and RF.

Besides, we display the time comparisons between KDB^*e*^ and RF in Figure 7. It is obvious that KDB^*e*^ enjoyed a great advantage over RF in terms of training time on datasets of all sizes. This advantage could be attributed to that KDB^*e*^ only learned a regular KDB for every dataset during the training phase while RF needed to train 100 decision trees. When comparing classification time, the performance of KDB^*e*^ and RF showed a slight reversal. Learning kdb^*e*^ for each unlabeled test instance made KDB^*e*^ take a bit more time than RF. However, when comparing on small and medium datasets, the advantage of RF over KDB^*e*^ was not significant. To conclude, on small and medium datasets, KDB^*e*^ had a significantly better zero-one loss performance and better RMSE than RF. This was packaged with KDB^*e*^’s far superior training times and competitive classification times over RF, which makes KDB^*e*^ an excellent alternative to RF, especially for dealing with small and medium datasets.

#### 4.3.1. Discussion

RF has been applied to several scientific fields and associated research areas [32], because of its high classification accuracy. However, RF is more negatively affected in terms of computation consumption (memory and time) by dataset sizes than BNCs [19]. Furthermore, due to the random mechanism, RF is sometimes criticized for difficulty giving a clear semantic explanation of the combined result that is outputted by numerous decision trees. In contrast, our proposed discriminatory target learning framework considers not only the dependence relationships that exist in the training data, but also that hidden in unlabeled test instances, which makes the final model highly interpretable. KDB^*e*^ outperformed RF in terms of zero-one loss, RMSE and variance, especially on small and medium size datasets, while RF beat KDB^*e*^ in terms of bias. Moreover, RF required substantially more time for training and KDB^*e*^ took a bit more time for classifying.

**Figure 8:**
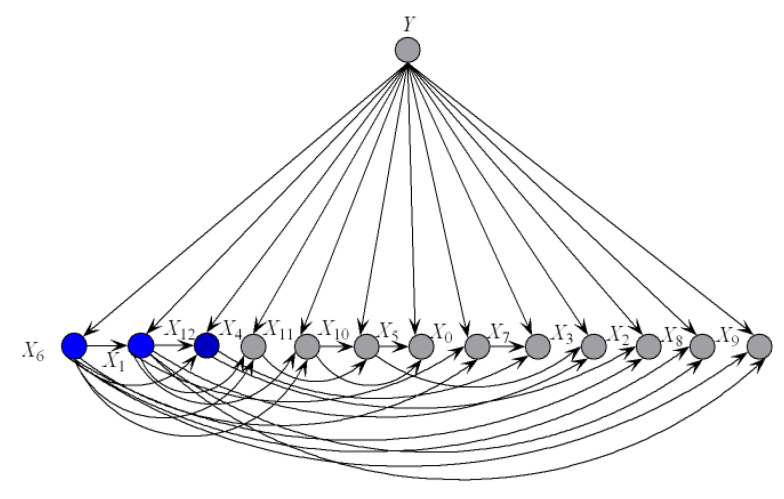
The structure of KDB on Heart-disease-c dataset.

**Figure 9:**
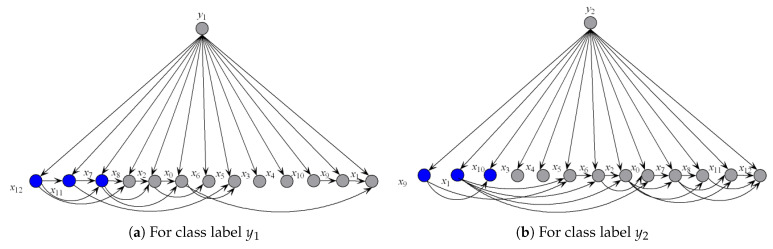
The structure of submodels of kdb^*e*^.

To illustrate the better interpretability of KDB^*e*^ than that of RF, we took medical diagnostic application as an example. The Heart-disease-c dataset (http://archive.ics.uci.edu/ml/datasets/Heart+Disease) from UCI Machine Learning Repository was collected from Cleveland Clinic Foundation, containing 13 attributes and two class labels. The detailed description of this dataset is shown in Table 7. The zero-one loss results of KDB, RF and KDB^*e*^ are 0.2244, 0.2212 and 0.2079, respectively. KDB learned from training data can describe the general conditional dependencies, while for a certain instance some of dependence relationships may hold instead of all the dependencies shown in KDB. In contrast, kdb^*e*^ can encode the most possible local conditional dependencies hidden in one single test instance. We argue that an ideal phenomenon is that KDB and kdb^*e*^ are complementary to each other for classification and they may focus on different key points. To prove this, randomly taking an instance from Heart-disease-c dataset as an example, the detail of this instance is shown as, $T=\{x_{0}=57,x_{1}=1,x_{2}=3,x_{3}=150,x_{4}=168,x_{5}=0,x_{6}=0,x_{7}=174,x_{8}=0,x_{9}=1.6,x_{10}=3,x_{11}=0,x_{12}=3\}$. Figure 8 and Figure 9 show the structural difference between KDB and the submodels of kdb^*e*^. For KDB, by comparing mutual information I(X;Y), $\left\{X_{6},X_{1},X_{12}\right\}$ are the first three key attributes for this dataset. There are 23 arcs in the structure of KDB which represent the conditional dependencies between predictive attributes. However, the values of $I(X_{8};X_{1}|Y)$, $I(X_{8};X_{6}|Y)$, $I(X_{9};X_{1}|Y)$ and $I(X_{9};X_{6}|Y)$ are all 0. For the instance T, in Figure 9, we can easily find that the structure of kdb^*e*^ differed greatly from that of KDB. The true class label for T is $y_{1}$. KDB misclassified T, while KDB^*e*^ correctly classified the instance. Thus, we can use Figure 9a for dependence analysis. By comparing the pointwise $y_{1}$-mutual information, $\left\{x_{12},x_{11},x_{7}\right\}$ are the first three key attribute values for T. It is worth mentioning that $X_{1}$ ranked second in KDB, whereas $x_{1}$ ranked last in kdb_*y*1_. Furthermore, there were only 15 arcs in kdb_*y*1_, which means that some redundant dependencies were eliminated. In general, KDB^*e*^ could utilize the knowledge learned from the training data and unlabeled test instances by building different models, which is obviously suitable for precision medical diagnosis.

#### 4.3.2. Imbalanced Datasets

There are 15 imbalanced datasets in our experiments, which are annotated with the symbol “*” in Table 1. To prove that KDB^*e*^ has the capacity to deal with imbalanced datasets, we conducted a set of experiments to compare the performance of KDB^*e*^ with RF in terms of extended Matthews correlation coefficient (MCC). The MCC provides a balanced measure for skewed datasets by taking into account the class distribution [33]. The classification results can be shown in the form of a confusion matrix as follows:

(13) $$\left[\begin{array}{ccc} N_{11} & \cdots \; & N_{1m}\\ \vdots & \ddots & \vdots \\ N_{m1} & \cdots \; & N_{mm} \end{array}\right]$$

Each entry $N_{ii}$ of the matrix gives the number of instances, whose true class was $Y_{i}$ that were actually assigned to $Y_{i}$, where 1≤i≤m. Each entry $N_{ij}$ of the matrix gives the number of instances, whose true class was $Y_{i}$ that were actually assigned to $Y_{j}$, where i≠j and 1≤i,j≤m. Given the confusion matrix, the extended MCC can be calculated as follow,

(14) $$MCC=\frac{\sum_{mij}N_{ii}N_{jm}-N_{ij}N_{mi}}{\sqrt{\sum_{i}\left(\sum_{j}N_{ij}\right)\left(\sum_{j^{\prime },i^{\prime }\neq i}N_{i^{\prime }j^{\prime }}\right)}\sqrt{\sum_{i}\left(\sum_{j}N_{ji}\right)\left(\sum_{j^{\prime },i^{\prime }\neq i}N_{j^{\prime }i^{\prime }}\right)}}$$

**Figure 10:**
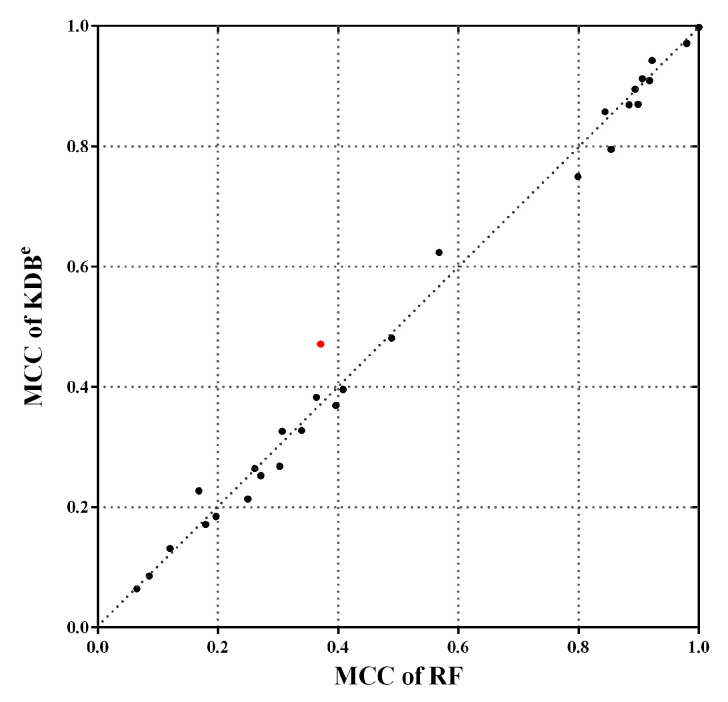
The scatter plot of KDB^*e*^ and RF in terms of MCC. Dis dataset is annotated with red color, which is a notable case where KDB^*e*^ enjoys significant advantages.

Note that the MCC reaches its best value at 1, which represents a perfect prediction, and worst value at −1, which indicates a total disagreement between the predicted and observed classifications. Figure 10 shows the scatter plot of KDB^*e*^ and RF in terms of MCC. We can see that many points fall close to the diagonal line, which means that KDB^*e*^ achieved competitive results compared with RF. Furthermore, there are three points far above the diagonal line, which means KDB^*e*^ enjoys significant advantages on these datasets. A notable case is Dis dataset annotated with red color, where the MCC of KDB^*e*^ (0.4714) was much higher than that of RF (0.3710). In general, KDB^*e*^ had the capacity to handle the imbalanced datasets.

### 4.4. Global Comparison of All Classifiers

**Figure 11:**
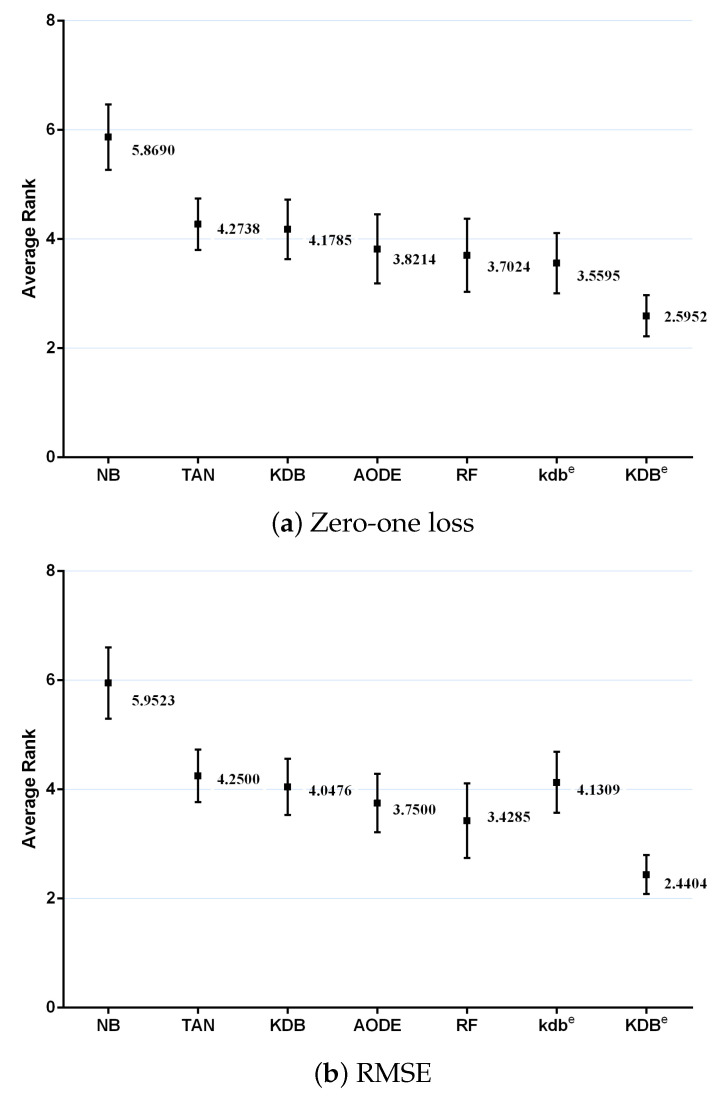
Average ranks in terms of zero-one loss and RMSE for all learners.

In this section, to assess whether the overall differences in performance of these learners was statistically significant, we employed the Friedman test [34] and the post-hoc Nemenyi test, as recommended by Demšar [35]. The Friedman test is a non-parametric test for multiple hypotheses testing. It ranks the algorithms for each dataset separately: the best performing algorithm getting the rank of 1, the second best ranking 2, and so on. In case of ties, average ranks are assigned. The null-hypothesis is that all of the algorithms perform almost equivalently and there is no significant difference in terms of average ranks. The Friedman statistic can be computed as follows:

(15) $$\chi_{F}^{2}=\frac{12}{Nt(t+1)}\sum_{j=1}^{t}R_{j}^{2}-3N\left(t+1\right),$$

where $R_{j}=\sum_{i}r_{i}^{j}$ and $r_{i}^{j}$ is the rank of the *j*th of *t* algorithms on the *i*th of *N* datasets. The Friedman statistic is distributed according to $\chi_{F}^{2}$ with t-1 degrees of freedom. Thus, for any pre-determined level of significance α, the null hypothesis will be rejected if $\chi_{F}^{2}>\chi_{\alpha }^{2}$. The critical value of $\chi_{\alpha }^{2}$ for α=0.05 with six degrees of freedom is 12.592. The Friedman statistics of zero-one loss and RMSE were 53.65 and 60.49, which were both larger than 12.592. Hence, the null-hypotheses was rejected. According to the detailed results of rank shown in Table A5 and Table A6 in Appendix A, Figure 11 plots the average ranks across all datasets, along with the standard deviation for each learner. When assessing the calibration of the probability estimates using zero-one loss, KDB^*e*^ obtained the lowest average rank of 2.5952, followed by kdb^*e*^ with 3.5595 and RF with 3.7024 (very close to those for AODE). When assessing performance using RMSE, KDB^*e*^ still performed the best, followed by RF with 3.4285 and AODE with 3.7500. We found NB at the other extreme on both measures, with average ranks 5.8690 and 5.9523 out of a total of seven learners.

**Figure 12:**
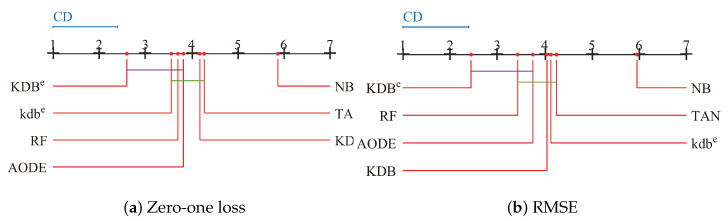
Nemenyi test in terms of zero-one loss and RMSE for all learners.

Since we rejected the null-hypotheses, Nemenyi test was used to further analyze which pairs of algorithms were significantly different in terms of average ranks of the Friedman test. The performance of two classifiers is significantly different if their corresponding average ranks of the Friedman test differ by at least the critical difference (CD):

(16) $$CD=q_{\alpha }\sqrt{\frac{t(t+1)}{6N}},$$

where the critical value $q_{\alpha }$ for α=0.05 and t=7 is 2.949. Given seven algorithms and 42 datasets, we used Equation (16) to calculate CD and the result is 1.3902. The learners in Figure 12 are plotted on the red line on the basis of their average ranks, corresponding to the nodes on the top black line. If two algorithms had no significant difference, they were connected by a line. As shown in Figure 12a, we easily found that KDB^*e*^ had a significantly lower average zero-one loss rank than NB, TAN and KDB. KDB^*e*^ also achieved lower average zero-one loss rank than kdb^*e*^, RF and AODE, but not significantly so. When RMSE was considered, KDB^*e*^ still performed the best and the rank of KDB^*e*^ was significantly lower than that of KDB, providing solid evidence for the effectiveness of our proposed discriminatory target learning framework.

## 5. Conclusions

Lack of explanatory insight into the relative influence of the random variables greatly restricts the application domain of machine learning techniques. By redefining mutual information and conditional information, the framework of discriminatory target learning can help fully and discriminately describe the dependency relationships in unlabeled instance and labeled training data. The kdb^*e*^ learned from unlabeled instance and regular KDB learned from training data are different but complementary in nature, which will help further improve the classification performance. Discriminatory target learning can be expected to play for different types of BNCs with different dependency complexities. Exploration of application of discriminatory target learning in other kinds of machine learning techniques, e.g., decision tree or support vector machine, is a further area for future work.

## Figures and Tables

**Table 1:** Datasets. Imbalanced datasets are annotated with the symbol “*”.

Index	Dataset	Instance	Attribute	Class	Index	Dataset	Instance	Attribute	Class
1	Contact-lenses	24	4	3	22	Kr-vs-kp	3196	36	2
2	Labor	57	16	2	23	Dis *	3772	29	2
3	Echocardiogram	131	6	2	24	Hypo	3772	29	4
4	Lymphography	148	18	4	25	Sick *	3772	29	2
5	Sonar	208	60	2	26	Abalone *	4177	8	3
6	Glass-id	214	9	3	27	Waveform-5000	5000	40	3
7	New-thyroid *	215	5	3	28	Phoneme	5438	7	50
8	Heart-disease-c	303	13	2	29	Wall-following	5456	24	4
9	Soybean-large	307	35	19	30	Page-blocks	5473	10	5
10	Ionosphere *	351	34	2	31	Satellite *	6435	36	6
11	Dermatology	366	34	6	32	Thyroid	9169	29	20
12	House-votes-84 *	435	16	2	33	Pendigits	10,992	16	10
13	Chess *	551	39	2	34	Sign	12,546	8	3
14	Soybean *	683	35	19	35	Nursery	12,960	8	5
15	Breast-cancer-w	699	9	2	36	Magic	19,020	10	2
16	Tic-tac-toe	958	9	2	37	Letter-recog	20,000	16	26
17	Vowel	990	13	11	38	Adult *	48,842	14	2
18	Car *	1728	6	4	39	Shuttle *	58,000	9	7
19	Mfeat-mor	2000	6	10	40	Connect-4	67,557	42	3
20	Segment	2310	19	7	41	Waveform *	100,000	21	3
21	Hypothyroid *	3163	25	2	42	Localization	164,860	5	11

**Table 2:** W/D/L comparison results of zero-one loss on all datasets.

	NB	TAN	KDB	AODE	kdb^*e*^
TAN	29/7/6	-	-	-	-
KDB	30/5/7	20/9/13	-	-	-
AODE	33/5/4	16/14/12	20/6/16	-	-
kdb^*e*^	30/5/7	17/18/7	20/11/11	13/15/14	-
KDB^*e*^	34/3/5	23/13/6	26/13/3	22/10/10	14/20/8

**Table 3:** W/D/L comparison results of RMSE on all datasets.

	NB	TAN	KDB	AODE	kdb^*e*^
TAN	32/4/6	-	-	-	-
KDB	32/4/6	16/19/7	-	-	-
AODE	29/9/4	16/19/7	15/15/12	-	-
kdb^*e*^	30/5/7	9/21/12	11/17/14	7/19/16	-
KDB^*e*^	34/3/5	20/19/3	17/20/5	17/17/8	21/21/0

**Table 4:** W/D/L comparison results of bias on all datasets.

	NB	TAN	KDB	AODE	kdb^*e*^
TAN	30/5/7	-	-	-	-
KDB	30/5/7	25/9/8	-	-	-
AODE	32/7/3	18/14/10	15/4/23	-	-
kdb^*e*^	31/3/8	20/10/12	15/8/19	16/11/15	-
KDB^*e*^	32/3/7	26/9/7	11/27/4	21/13/8	17/18/7

**Table 5:** W/D/L comparison results of variance on all datasets.

	NB	TAN	KDB	AODE	kdb^*e*^
TAN	4/3/35	-	-	-	-
KDB	8/1/33	9/7/26	-	-	-
AODE	9/8/25	30/8/4	34/3/5	-	-
kdb^*e*^	7/1/34	19/13/10	30/4/8	6/10/26	-
KDB^*e*^	8/2/32	16/12/14	34/8/0	7/4/31	12/9/21

**Table 6:** W/D/L records between KDB^*e*^ and RF.

	All	Small	Medium	Large
Zero-one loss	20/10/12	10/4/3	7/4/4	3/2/5
RMSE	16/15/11	4/9/4	8/4/3	4/2/4
Bias	11/11/20	5/1/11	5/5/5	1/5/4
Variance	26/4/12	11/3/3	7/1/7	8/0/2

**Table 7:** Description of Heart-disease-c dataset.

Attribute	Description	Symbol
age	real value	$X_{0}$
sex	male or female, {0,1}	$X_{1}$
cp	chest pain type (angina, abnang, notang, asympt), {1,2,3,4}	$X_{2}$
trestbps	resting blood pressure, real value	$X_{3}$
chol	cholesterol, real value	$X_{4}$
fbs	fasting blood sugar < 120 (true or false), {0,1}	$X_{5}$
restecg	resting electrocardiographic results (norm, abn, hyper), {0,1,2}	$X_{6}$
thalach	maximum heart rate achieved, real value	$X_{7}$
exang	exercise induced angina (true or false), {0,1}	$X_{8}$
oldpeak	ST depression induced by exercise relative to rest, real value	$X_{9}$
slope	the slope of the peak exercise ST segment (up, flat, down), {1,2,3}	$X_{10}$
ca	number of vessels colored, real value	$X_{11}$
thal	thal (norm, fixed, rever), {3,6,7}	$X_{12}$
class	0 for health, 1 for sick	*Y*

## Appendix A. Tables of the Experimental Section

The best results in each row of each table are annotated with bold font.

**Table A1:** Experimental results of average zero-one loss.

Dataset	NB	TAN	KDB	AODE	RF	kdb^*e*^	KDB^*e*^
Contact-lenses	0.3750	0.3750	**0.2500**	0.3750	0.3438	0.3750	0.2917
Labor	**0.0351**	0.0526	**0.0351**	0.0526	0.0939	**0.0351**	**0.0351**
Echocardiogram	0.3359	0.3282	0.3435	0.3206	0.3489	**0.3130**	**0.3130**
Lymphography	**0.1486**	0.1757	0.2365	0.1689	0.2132	0.1757	0.2095
Sonar	0.2308	0.2212	0.2452	0.2260	**0.2067**	0.2452	0.2308
Glass-id	0.2617	0.2196	0.2196	0.2523	**0.2132**	0.2243	0.2150
New-thyroid	0.0512	0.0651	0.0698	**0.0465**	0.0816	0.0605	0.0605
Heart-disease-c	**0.1815**	0.2079	0.2244	0.2013	0.2212	0.1947	0.2079
Soybean-large	0.1238	0.1107	0.0879	**0.0782**	0.1107	0.1270	0.0814
Ionosphere	0.1054	0.0684	0.0741	0.0741	0.0766	0.0912	**0.0655**
Dermatology	0.0191	0.0328	0.0656	**0.0164**	0.0367	0.0546	0.0519
House-votes-84	0.0943	0.0552	0.0506	0.0529	**0.0416**	0.0575	0.0437
Chess	0.1125	**0.0926**	0.0998	0.0998	0.1074	**0.0926**	**0.0926**
Soybean	0.0893	**0.0469**	0.0556	**0.0469**	0.0703	0.0542	0.0527
Breast-cancer-w	**0.0258**	0.0415	0.0744	0.0358	0.0386	0.0401	0.0629
Tic-tac-toe	0.3069	0.2286	0.2035	0.2651	0.2115	**0.1931**	0.2004
Vowel	0.4242	**0.1303**	0.1818	0.1495	0.1674	0.1788	0.1626
Car	0.1400	0.0567	**0.0382**	0.0816	0.0772	0.0596	0.0411
Mfeat-mor	0.3140	**0.2970**	0.3060	0.3145	0.3000	0.3015	0.3035
Segment	0.0788	0.0390	0.0472	**0.0342**	0.0413	0.0355	0.0433
Hypothyroid	0.0149	0.0104	0.0107	0.0136	0.0122	**0.0092**	0.0095
Kr-vs-kp	0.1214	0.0776	0.0416	0.0842	**0.0128**	0.0460	0.0382
Dis	0.0159	0.0159	0.0138	0.0130	0.0133	0.0127	**0.0122**
Hypo	0.0138	0.0141	0.0114	**0.0095**	0.0122	0.0098	0.0098
Sick	0.0308	0.0257	**0.0223**	0.0273	0.0263	0.0270	0.0233
Abalone	0.4762	0.4587	0.4563	**0.4472**	0.4823	0.4534	0.4484
Waveform-5000	0.2006	0.1844	0.2000	**0.1462**	0.1558	0.1782	0.1756
Phoneme	0.2615	0.2733	0.1984	0.2392	**0.1789**	0.3139	0.1931
Wall-following	0.1054	0.0554	0.0401	0.0370	**0.0216**	0.0398	0.0387
Page-blocks	0.0619	0.0415	0.0391	0.0338	**0.0309**	0.0323	0.0322
Satellite	0.1806	0.1214	0.1080	0.1148	0.1085	0.1265	**0.1052**
Thyroid	0.1111	0.0720	0.0706	0.0701	0.0750	**0.0586**	0.0642
Pendigits	0.1181	0.0321	0.0294	**0.0200**	0.0339	0.0202	0.0248
Sign	0.3586	0.2755	0.2539	0.2821	**0.2038**	0.2685	0.2419
Nursery	0.0973	0.0654	0.0289	0.0730	**0.0248**	0.0509	0.0356
Magic	0.2239	0.1675	0.1637	0.1752	0.1674	0.1716	**0.1598**
Letter-recog	0.2525	0.1300	0.0986	0.0883	0.0902	**0.0675**	0.0861
Adult	0.1592	0.1380	0.1383	0.1493	**0.1204**	0.1315	0.1316
Shuttle	0.0039	0.0015	0.0009	0.0008	**0.0005**	0.0006	0.0007
Connect-4	0.2783	0.2354	0.2283	0.2420	**0.1875**	0.2337	0.2268
Waveform	0.0220	0.0202	0.0256	**0.0180**	0.1558	0.0194	0.0193
Localization	0.4955	0.3575	0.2964	0.3596	0.2976	**0.2659**	0.2743

**Table A2:** Experimental results of average RMSE.

Dataset	NB	TAN	KDB	AODE	RF	kdb^*e*^	KDB^*e*^
Contact-lenses	0.3778	0.4496	**0.3639**	0.4066	0.4098	0.4086	0.3825
Labor	**0.1420**	0.2185	0.1685	0.1900	0.2824	0.3647	0.2271
Echocardiogram	0.4896	0.4886	0.4889	0.4903	**0.4574**	0.4782	0.4813
Lymphography	**0.2446**	0.2684	0.3031	0.2478	0.2701	0.2729	0.2680
Sonar	0.4421	0.4131	0.4084	0.4285	**0.3518**	0.4071	0.3959
Glass-id	0.3540	0.3332	0.3395	0.3439	**0.3146**	0.3311	0.3275
New-thyroid	**0.1544**	0.1731	0.1797	0.1614	0.1560	0.1689	0.1714
Heart-disease-c	0.3743	0.3775	0.3963	0.3659	0.3696	**0.3572**	0.3690
Soybean-large	0.1032	0.0963	0.0858	0.0858	0.1143	0.1051	**0.0856**
Ionosphere	0.3157	0.2615	0.2714	0.2506	**0.2403**	0.2822	0.2523
Dermatology	**0.0631**	0.0851	0.1206	0.0692	0.1303	0.1857	0.1313
House-votes-84	0.2997	0.2181	0.1969	0.1994	**0.1846**	0.1962	0.1847
Chess	0.2944	**0.2594**	0.2615	0.2725	0.2771	0.2937	0.2642
Soybean	0.0933	**0.0642**	0.0654	0.0656	0.0922	0.0754	0.0643
Breast-cancer-w	**0.1570**	0.1928	0.2497	0.1848	0.1796	0.2194	0.2137
Tic-tac-toe	0.4309	0.4023	0.3772	0.3995	**0.2916**	0.3830	0.3693
Vowel	0.2270	**0.1271**	0.1582	0.1425	0.1581	0.1685	0.1516
Car	0.2252	0.1617	**0.1379**	0.2005	0.1782	0.1749	0.1505
Mfeat-mor	0.2086	**0.1940**	0.1974	0.1985	0.2074	0.1948	0.1954
Segment	0.1398	0.0967	0.1034	**0.0879**	0.1061	0.0957	0.0919
Hypothyroid	0.1138	0.0955	0.0937	0.1036	**0.0770**	0.0979	0.0913
Kr-vs-kp	0.3022	0.2358	0.1869	0.2638	**0.1268**	0.2626	0.2091
Dis	0.1177	0.1103	0.1024	0.1080	**0.1011**	0.1074	0.1021
Hypo	0.0766	0.0738	0.0671	0.0650	0.0715	0.0719	**0.0635**
Sick	0.1700	0.1434	**0.1382**	0.1572	0.1487	0.1489	0.1394
Abalone	0.4630	0.4250	0.4277	**0.4193**	0.4539	0.4220	0.4220
Waveform-5000	0.3348	0.2947	0.3149	**0.2659**	0.3036	0.2950	0.2869
Phoneme	0.0880	0.0902	0.0784	0.0885	**0.0731**	0.0952	0.0783
Wall-following	0.2177	0.1586	0.1363	0.1292	**0.1206**	0.1315	0.1210
Page-blocks	0.1450	0.1187	0.1128	0.1021	0.0974	**0.0972**	0.0991
Satellite	0.2400	0.1851	0.1777	0.1800	0.1682	0.1865	**0.1644**
Thyroid	0.0967	0.0746	0.0744	0.0745	0.0770	**0.0674**	0.0679
Pendigits	0.1427	0.0725	0.0687	**0.0568**	0.0979	0.0793	0.0646
Sign	0.3984	0.3505	0.3334	0.3524	**0.3104**	0.3468	0.3300
Nursery	0.1766	0.1385	0.1121	0.1571	**0.1010**	0.1372	0.1217
Magic	0.3974	0.3461	0.3470	0.3541	0.3571	0.3514	**0.3411**
Letter-recog	0.1184	0.0860	0.0768	0.0707	0.0896	0.0756	**0.0685**
Adult	0.3409	0.3076	0.3089	0.3245	0.3274	0.3021	**0.3015**
Shuttle	0.0298	0.0182	0.0146	0.0126	0.0142	**0.0121**	0.0125
Connect-4	0.3587	0.3315	0.3247	0.3370	**0.3057**	0.3409	0.3279
Waveform	0.1176	0.0951	0.1145	0.0860	**0.0799**	0.0999	0.0901
Localization	0.2390	0.2095	0.1960	0.2095	0.1939	**0.1834**	0.1846

**Table A3:** Experimental results of average bias.

Dataset	NB	TAN	KDB	AODE	RF	kdb^*e*^	KDB^*e*^
Contact-lenses	0.2163	0.1825	0.3175	0.2850	**0.1748**	0.1863	0.2850
Labor	0.0289	0.0211	0.0279	0.0347	0.0409	**0.0184**	0.0279
Echocardiogram	0.2844	0.2642	0.3065	0.2751	**0.2256**	0.2602	0.2686
Lymphography	**0.0902**	0.1027	0.1041	0.0933	0.1288	0.0951	0.0996
Sonar	0.1672	0.1646	0.1686	0.1696	**0.1045**	0.1829	0.1762
Glass-id	0.2901	0.2756	0.2713	0.2785	**0.1348**	0.2730	0.2732
New-thyroid	0.0290	**0.0277**	0.0348	**0.0277**	0.0285	0.0279	0.0396
Heart-disease-c	0.1297	0.1263	0.1299	0.1138	0.1304	**0.1128**	0.1274
Soybean-large	0.1070	0.1422	0.1086	**0.0648**	0.1213	0.1717	0.1112
Ionosphere	0.1220	0.0804	0.0855	0.0744	**0.0624**	0.0912	0.0862
Dermatology	0.0079	0.0274	0.0489	**0.0055**	0.0190	0.0541	0.0451
House-votes-84	0.0899	0.0410	**0.0258**	0.0430	0.0327	0.0457	0.0301
Chess	0.1413	0.1437	0.1119	0.1290	**0.0548**	0.1265	0.1192
Soybean	0.1015	0.0522	**0.0491**	0.0524	0.0586	0.0971	0.0502
Breast-cancer-w	**0.0187**	0.0384	0.0449	0.0338	0.0301	0.0221	0.0348
Tic-tac-toe	0.2614	0.1746	**0.1367**	0.2005	0.0270	0.1434	0.1390
Vowel	0.3301	0.1942	0.1745	0.1895	**0.0756**	0.1845	0.1736
Car	0.0937	0.0478	0.0387	0.0556	0.0389	0.0389	**0.0374**
Mfeat-mor	0.2624	**0.2077**	0.2142	0.2477	0.2311	0.2223	0.2166
Segment	0.0785	0.0491	0.0453	0.0367	**0.0253**	0.0387	0.0419
Hypothyroid	0.0116	0.0104	0.0096	0.0094	0.0516	**0.0090**	0.0094
Kr-vs-kp	0.1107	0.0702	0.0417	0.0747	**0.0063**	0.0434	0.0407
Dis	**0.0165**	0.0193	0.0191	0.0170	0.0203	0.0192	0.0191
Hypo	0.0092	0.0124	0.0077	**0.0071**	0.0083	0.0098	0.0073
Sick	0.0246	0.0207	0.0198	0.0224	**0.0194**	0.0254	0.0196
Abalone	0.4180	0.3126	**0.3033**	0.3201	0.3257	0.3195	0.3132
Waveform-5000	0.1762	0.1232	0.1157	0.1235	**0.1114**	0.1219	0.1147
Phoneme	0.2216	0.2394	0.1572	0.2207	**0.1102**	0.2927	0.1551
Wall-following	0.0951	0.0491	0.0257	0.0251	**0.0122**	0.0296	0.0245
Page-blocks	0.0451	0.0308	0.0280	0.0251	**0.0217**	0.0277	0.0264
Satellite	0.1746	0.0950	0.0808	0.0902	0.0874	0.1011	**0.0802**
Thyroid	0.0994	0.0587	0.0553	0.0611	0.0516	**0.0493**	0.0531
Pendigits	0.1095	0.0314	0.0207	0.0228	0.0216	0.0196	**0.0189**
Sign	0.3257	0.2420	0.2161	0.2531	**0.1540**	0.2322	0.2132
Nursery	0.0928	0.0521	0.0281	0.0651	**0.0086**	0.0400	0.0322
Magic	0.2111	0.1252	**0.1241**	0.1600	0.1244	0.1323	0.1265
Letter-recog	0.2207	0.1032	0.0806	0.0876	**0.0490**	0.0700	0.0732
Adult	0.1649	0.1312	0.1220	0.1437	**0.1109**	0.1240	0.1226
Shuttle	0.0040	0.0008	0.0007	**0.0006**	**0.0006**	**0.0006**	**0.0006**
Connect-4	0.2660	0.2253	0.2022	0.2264	**0.1427**	0.2169	0.2075
Waveform	0.0219	**0.0152**	0.0210	0.0156	0.0158	0.0172	0.0161
Localization	0.4523	0.3106	0.2134	0.3129	0.2047	**0.2027**	0.2038

**Table A4:** Experimental results of average variance.

Dataset	NB	TAN	KDB	AODE	RF	kdb^*e*^	KDB^*e*^
Contact-lenses	0.1713	0.1925	0.1700	**0.1275**	0.2013	0.2138	0.1775
Labor	0.0395	0.0632	0.0721	**0.0179**	0.0758	0.0605	0.0721
Echocardiogram	0.1272	**0.1265**	0.1400	0.1319	0.1469	0.1374	0.1337
Lymphography	**0.0343**	0.1116	0.1408	0.0476	0.1352	0.0927	0.1249
Sonar	**0.0907**	0.1165	0.1199	0.0942	0.1189	0.0983	0.1107
Glass-id	**0.0930**	0.1075	0.1189	0.1004	0.1089	0.1101	0.1099
New-thyroid	**0.0161**	0.0272	0.0385	0.0230	0.0365	0.0285	0.0351
Heart-disease-c	**0.0248**	0.0479	0.0582	0.0357	0.0718	0.0466	0.0498
Soybean-large	**0.0783**	0.1176	0.0982	0.0842	0.1373	0.0921	0.0947
Ionosphere	**0.0242**	0.0401	0.0581	0.0385	0.0582	0.0344	0.0497
Dermatology	0.0216	0.0513	0.0684	**0.0199**	0.0685	0.0746	0.0648
House-votes-84	**0.0066**	0.0170	0.0197	0.0094	0.0179	0.0164	0.0168
Chess	**0.0401**	0.0486	0.0531	0.0415	0.0626	0.0423	0.0447
Soybean	**0.0302**	0.0654	0.0439	0.0326	0.0606	0.0509	0.0406
Breast-cancer-w	**0.0010**	0.0337	0.0504	0.0134	0.0101	0.0199	0.0425
Tic-tac-toe	**0.0455**	0.0824	0.1125	0.0513	0.0590	0.0813	0.0951
Vowel	0.2542	0.2445	0.2325	0.2344	**0.1093**	0.2337	0.2255
Car	0.0520	**0.0376**	0.0434	0.0438	0.0456	0.0447	0.0379
Mfeat-mor	**0.0622**	0.1020	0.1031	0.0677	0.1351	0.0882	0.0960
Segment	0.0259	0.0294	0.0381	0.0255	**0.0191**	0.0291	0.0344
Hypothyroid	0.0031	0.0034	**0.0024**	0.0034	0.0279	0.0034	**0.0024**
Kr-vs-kp	0.0186	0.0152	0.0111	0.0186	0.0077	**0.0076**	0.0077
Dis	0.0069	0.0005	0.0011	0.0071	0.0021	0.0005	**0.0003**
Hypo	0.0051	0.0071	0.0069	0.0049	**0.0046**	0.0078	0.0060
Sick	0.0047	0.0051	0.0043	0.0042	0.0082	0.0052	**0.0035**
Abalone	**0.0682**	0.1693	0.1769	0.1544	0.1865	0.1511	0.1633
Waveform-5000	**0.0259**	0.0690	0.0843	0.0410	0.0528	0.0625	0.0666
Phoneme	0.1215	0.1828	0.1064	0.1343	**0.0818**	0.1850	0.1052
Wall-following	0.0211	0.0288	0.0294	0.0242	**0.0112**	0.0266	0.0278
Page-blocks	0.0135	0.0143	0.0177	0.0124	**0.0110**	0.0115	0.0146
Satellite	**0.0139**	0.0367	0.0455	0.0363	0.0251	0.0388	0.0406
Thyroid	**0.0205**	0.0257	0.0272	0.0235	0.0279	0.0220	0.0235
Pendigits	0.0157	0.0200	0.0236	**0.0127**	0.0148	0.0157	0.0198
Sign	**0.0313**	0.0386	0.0596	0.0378	0.0593	0.0572	0.0488
Nursery	**0.0085**	0.0168	0.0195	0.0105	0.0193	0.0179	0.0168
Magic	**0.0174**	0.0490	0.0491	0.0297	0.0512	0.0407	0.0440
Letter-recog	0.0471	0.0591	0.0709	0.0448	0.0492	**0.0440**	0.0619
Adult	**0.0069**	0.0165	0.0285	0.0116	0.0425	0.0141	0.0185
Shuttle	0.0009	0.0004	**0.0003**	0.0004	0.0004	0.0004	**0.0003**
Connect-4	0.0156	**0.0149**	0.0309	0.0222	0.0534	0.0215	0.0222
Waveform	**0.0009**	0.0053	0.0037	0.0025	0.0068	0.0021	0.0035
Localization	**0.0460**	0.0594	0.1099	0.0580	0.1106	0.0897	0.0955

**Table A5:** Ranks in terms of zero-one loss of different learners.

Dataset	NB	TAN	KDB	AODE	RF	kdb^*e*^	KDB^*e*^
Contact-lenses	5.0	5.0	**1.0**	7.0	3.0	5.0	2.0
Labor	**2.5**	5.0	**2.5**	6.0	7.0	**2.5**	**2.5**
Echocardiogram	5.0	4.0	6.0	3.0	7.0	**1.5**	**1.5**
Lymphography	**1.0**	3.5	7.0	2.0	6.0	3.5	5.0
Sonar	4.5	2.0	6.5	3.0	**1.0**	6.5	4.5
Glass-id	7.0	3.5	3.5	6.0	**1.0**	5.0	2.0
New-thyroid	2.0	5.0	6.0	**1.0**	7.0	3.5	3.5
Heart-disease-c	**1.0**	4.5	7.0	3.0	6.0	2.0	4.5
Soybean-large	6.0	4.5	3.0	**1.0**	4.5	7.0	2.0
Ionosphere	7.0	2.0	3.0	4.0	5.0	6.0	**1.0**
Dermatology	2.0	3.0	7.0	**1.0**	4.0	6.0	5.0
House-votes-84	7.0	5.0	3.0	4.0	**1.0**	6.0	2.0
Chess	7.0	2.0	4.0	5.0	6.0	**2.0**	**2.0**
Soybean	7.0	**1.0**	5.0	2.0	6.0	4.0	3.0
Breast-cancer-w	**1.0**	5.0	7.0	2.0	3.0	4.0	6.0
Tic-tac-toe	7.0	5.0	3.0	6.0	4.0	**1.0**	2.0
Vowel	7.0	**1.0**	6.0	2.0	4.0	5.0	3.0
Car	7.0	3.0	**1.0**	6.0	5.0	4.0	2.0
Mfeat-mor	6.0	**1.0**	5.0	7.0	2.0	3.0	4.0
Segment	7.0	3.0	6.0	**1.0**	4.0	2.0	5.0
Hypothyroid	7.0	3.0	4.0	6.0	5.0	**1.0**	2.0
Kr-vs-kp	7.0	5.0	3.0	6.0	**1.0**	4.0	2.0
Dis	6.5	6.5	5.0	3.0	4.0	2.0	**1.0**
Hypo	6.0	7.0	4.0	**1.0**	5.0	2.5	2.5
Sick	7.0	3.0	**1.0**	6.0	4.0	5.0	2.0
Abalone	6.0	5.0	4.0	2.0	7.0	3.0	**1.0**
Waveform-5000	7.0	5.0	6.0	**1.0**	2.0	4.0	3.0
Phoneme	5.0	6.0	3.0	4.0	**1.0**	7.0	2.0
Wall-following	7.0	6.0	5.0	2.0	**1.0**	4.0	3.0
Page-blocks	7.0	6.0	5.0	4.0	**1.0**	3.0	2.0
Satellite	7.0	5.0	2.0	4.0	3.0	6.0	**1.0**
Thyroid	7.0	5.0	3.0	4.0	6.0	**1.0**	2.0
Pendigits	7.0	5.0	4.0	**1.5**	6.0	**1.5**	3.0
Sign	7.0	5.0	3.0	6.0	**1.0**	4.0	2.0
Nursery	7.0	5.0	2.0	6.0	**1.0**	4.0	3.0
Magic	7.0	4.0	2.0	6.0	3.0	5.0	**1.0**
Letter-recog	7.0	6.0	5.0	3.0	4.0	**1.0**	2.0
Adult	7.0	4.0	5.0	6.0	**1.0**	2.0	3.0
Shuttle	7.0	6.0	5.0	4.0	**1.0**	2.0	3.0
Connect-4	7.0	5.0	3.0	6.0	**1.0**	4.0	2.0
Waveform	5.0	4.0	6.0	**1.0**	7.0	3.0	2.0
Localization	7.0	5.0	3.0	6.0	4.0	**1.0**	2.0
Sum of ranks	246.5	179.5	175.5	160.5	155.5	149.5	**109.0**

**Table A6:** Ranks in terms of RMSE of different learners.

Dataset	NB	TAN	KDB	AODE	RF	kdb^*e*^	KDB^*e*^
Contact-lenses	2.0	7.0	**1.0**	4.0	6.0	5.0	3.0
Labor	**1.0**	4.0	2.0	3.0	6.0	7.0	5.0
Echocardiogram	5.0	4.0	7.0	6.0	**1.0**	2.0	3.0
Lymphography	**1.0**	4.0	7.0	2.0	5.0	6.0	3.0
Sonar	7.0	5.0	4.0	6.0	**1.0**	3.0	2.0
Glass-id	7.0	4.0	5.0	6.0	**1.0**	3.0	2.0
New-thyroid	**1.0**	6.0	7.0	3.0	2.0	4.0	5.0
Heart-disease-c	5.0	6.0	7.0	2.0	4.0	**1.0**	3.0
Soybean-large	5.0	4.0	3.0	2.0	7.0	6.0	**1.0**
Ionosphere	7.0	4.0	5.0	2.0	**1.0**	6.0	3.0
Dermatology	**1.0**	3.0	4.0	2.0	5.0	7.0	6.0
House-votes-84	7.0	6.0	4.0	5.0	**1.0**	3.0	2.0
Chess	7.0	**1.0**	2.0	4.0	5.0	6.0	3.0
Soybean	7.0	**1.0**	4.0	3.0	6.0	5.0	2.0
Breast-cancer-w	**1.0**	4.0	7.0	3.0	2.0	6.0	5.0
Tic-tac-toe	7.0	6.0	3.0	5.0	**1.0**	4.0	2.0
Vowel	7.0	**1.0**	5.0	2.0	4.0	6.0	3.0
Car	7.0	3.0	**1.0**	6.0	5.0	4.0	2.0
Mfeat-mor	7.0	**1.0**	5.0	4.0	6.0	2.0	3.0
Segment	7.0	4.0	5.0	**1.0**	6.0	3.0	2.0
Hypothyroid	7.0	4.0	3.0	6.0	**1.0**	5.0	2.0
Kr-vs-kp	7.0	4.0	2.0	6.0	**1.0**	5.0	3.0
Dis	7.0	6.0	3.0	5.0	**1.0**	4.0	2.0
Hypo	7.0	6.0	3.0	2.0	4.0	5.0	**1.0**
Sick	7.0	3.0	2.0	6.0	4.0	5.0	**1.0**
Abalone	7.0	4.0	5.0	**1.0**	6.0	2.5	2.5
Waveform-5000	7.0	3.0	6.0	**1.0**	5.0	4.0	2.0
Phoneme	4.0	6.0	3.0	5.0	**1.0**	7.0	2.0
Wall-following	7.0	6.0	5.0	3.0	**1.0**	4.0	2.0
Page-blocks	7.0	6.0	5.0	4.0	2.0	**1.0**	3.0
Satellite	7.0	5.0	3.0	4.0	2.0	6.0	**1.0**
Thyroid	7.0	4.0	5.0	3.0	6.0	**1.0**	2.0
Pendigits	7.0	4.0	3.0	**1.0**	6.0	5.0	2.0
Sign	7.0	5.0	3.0	6.0	**1.0**	4.0	2.0
Nursery	7.0	5.0	2.0	6.0	**1.0**	4.0	3.0
Magic	7.0	2.0	3.0	5.0	6.0	4.0	**1.0**
Letter-recog	7.0	5.0	4.0	2.0	6.0	3.0	**1.0**
Adult	7.0	3.0	4.0	5.0	6.0	2.0	**1.0**
Shuttle	7.0	6.0	5.0	3.0	4.0	**1.0**	2.0
Connect-4	7.0	4.0	3.0	5.0	**1.0**	6.0	2.0
Waveform	7.0	4.0	6.0	2.0	**1.0**	5.0	3.0
Localization	7.0	5.5	4.0	5.5	3.0	**1.0**	2.0
Sum of ranks	250.0	178.5	170.0	157.5	144.0	173.5	**102.5**
